# Construction of enhanced transcriptional activators for improving cellulase production in *Trichoderma reesei* RUT C30

**DOI:** 10.1186/s40643-018-0226-4

**Published:** 2018-08-18

**Authors:** Jiajia Zhang, Chuan Wu, Wei Wang, Wei Wang, Dongzhi Wei

**Affiliations:** 1 New World Institute of Biotechnology, State Key Lab of Bioreactor Engineering, East China University of Science and Technology, P.O.B. 311, 130 Meilong Road, Shanghai, 200237 China; 2 State Key Lab of Bioreactor Engineering, East China University of Science and Technology, Shanghai, 200237 China

**Keywords:** Enhanced transcriptional activator, *Trichoderma reesei*, Transcription factor, Cellulase, XYR1, ACE2, ACE1

## Abstract

Enhancing cellulase production in *Trichoderma reesei* is of great interest for an economical biorefinery. Artificial transcription factors are a potentially powerful molecular strategy for improving cellulase production in *T. reesei*. In this study, enhanced transcriptional activators XYR1VP, ACE2VP, and ACE1VP were constructed by linking the C terminus of XYR1, ACE2, or ACE1 with an activation domain of herpes simplex virus protein VP16. *T. reesei* transformants T_XYR1VP_, T_ACE2VP_, and T_ACE1VP_ showed improved cellulase and/or xylanase production. T_XYR1VP_ has a cellulase-free phenotype but with significantly elevated xylanase production. Xylanase I and xylanase II activities [U/(mg biomass)] increased by 51% and 80%, respectively, in T_XYR1VP_ in comparison with parental strain RUT C30. The filter paper activity of T_ACE2VP_ in the Avicel-based medium increased by 52% compared to that of RUT C30. In the Avicel-based medium, T_ACE1VP_ manifested an 80% increase in FPase activity and a 50% increase in xylanase activity as compared to those of RUT C30. Additionally, when pretreated corn stover was hydrolyzed, crude enzymes produced from T_ACE1VP_ yielded a greater glucose release than did the enzymes produced by parental strain RUT C30.

**Electronic supplementary material:**

The online version of this article (10.1186/s40643-018-0226-4) contains supplementary material, which is available to authorized users.

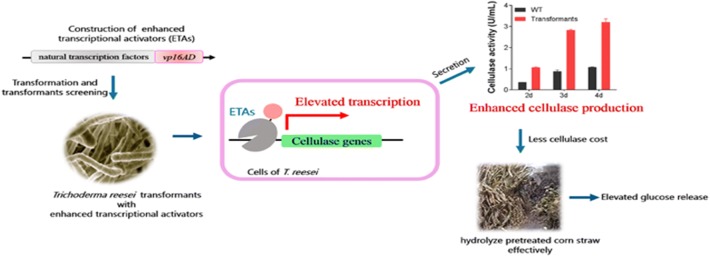

## Background

Production of environment-friendly biofuels and chemicals from lignocellulosic biomass, which forms the skeleton of all plant cells, has received extensive attention (Hahn-Hagerdal et al. 2006). The conversion of lignocellulosic biomass into monosaccharides using cellulase is a critical step in the biorefinery process (Parisutham et al. 2014). The enhancement of cellulase production greatly reduces the cost of a biorefinery of lignocellulosic biomass (Vicari et al. 2012; Biddy et al. 2016).

*Trichoderma reesei*, an excellent secretor of enzymes, has been widely used for the industrial production of cellulase (Bischof et al. 2016). *T. reesei* produces three types of cellulases: cellobiohydrolases (CBH1 and CBH2), endoglucanases (mainly include EGL1 and EGL2), and β-glucosidase (mainly BGL1). Cellobiohydrolases and endoglucanases synergistically hydrolyze cellulose to produce cello-oligosaccharides (mainly cellobiose). β-Glucosidases degrade cellobiose into the end product glucose. *T. reesei* also expresses large amounts of xylanase, among which XYN1 and XYN2 are the most abundant (Zeilinger et al. 1996). Strain RUT C30 serves as a cellulase hyperproducer and is employed in research and in the industry (Martinez et al. 2008; Bischof et al. 2016). Therefore, enhancing cellulase production in *T. reesei* RUT C30 is useful for an economical biorefinery.

Cellulase production in *T. reesei* RUT C30 is transcriptionally coregulated by a set of transcription factors (TFs), including XYR1, ACE3, ACE2, and ACE1 (Mach-Aigner et al. 2008; Häkkinen et al. 2014; Bischof et al. 2016). Among them, XYR1 and ACE3 are key transcriptional activators, and deletion of *xyr1* or *ace3* abrogates cellulase production (Stricker et al. 2006; Akel et al. 2009; Häkkinen et al. 2014; Castro Santos et al. 2016). ACE2 also serves as a transcriptional activator of cellulase production, and deletion of *ace2* decreases mRNA levels of cellulase-encoding genes, such as *cbh1*, *cbh2*, *egl1*, and *egl2* (Aro et al. 2001). ACE1 is recognized as a transcriptional repressor of cellulase production, and deletion of *ace1* increased the production of all main cellulase and xylanase in sophorose- and cellulose-induced cultures (Saloheimo et al. 2000; Aro et al. 2003; Portnoy et al. 2011).

Artificial TFs are a potentially powerful molecular strategy for modulating target gene expression and for obtaining an enhanced phenotype (Ju et al. 2008; Lee et al. 2013). Specific artificial transcriptional activators have been constructed to improve cellulase production in *T. reesei* (Su et al. 2009; Zhang et al. 2016a, 2017, 2018). An artificial transcriptional activator constructed by fusing the two DNA-binding domains of ACE1 and CRE1 with an effector domain of ACE2 can regulate the expression of cellulase genes (Su et al. 2009). *T. reesei* U3, a mutant strain with enhanced cellulase production, was identified via screening with construction of an artificial zinc finger protein library (Zhang et al. 2016a). Similarly, transformant zxy-2 of an artificial transcriptional activator—containing the binding domain of CRE1 linked to the effector and binding domains of XYR1—yields constitutive cellulase production from glucose as the sole carbon source (Zhang et al. 2017). A universal and simple pattern strategy for constructing a series of artificial transcriptional activators in *T. reesei* must be developed further. Recently, we improved cellulase production in *T. reesei* by substituting natural TFs with minimal transcriptional activators (Zhang et al. 2018) designed by linking one DNA-binding domain of ACE2 or CRE1 to the C-terminal 78 amino acid residues (aa) of herpes simplex virus protein VP16, which act as an activation domain that activates transcription of early viral genes (Sadowski et al. 1988; Triezenberg et al. 1988).

Here, we developed three novel artificial transcriptional activators as enhanced transcriptional activators (ETAs)—XYR1VP, ACE2VP, and ACE1VP—in *T. reesei* RUT C30 via a universal and simple pattern strategy. These ETAs were constructed by fusing the strong transcriptional activation domain of VP16 to the C terminus of natural TFs (XYR1, ACE2, and ACE1) and transfected into hypercellulolytic strain *T. reesei* RUT C30 to replace the natural TFs (XYR1, ACE2, and ACE1, respectively) by homologous double exchange. Next, the effects of these ETAs on cellulase production were investigated. These ETAs were shown to improve cellulase production in *T. reesei*. Our study offers a novel strategy for obtaining high-yield cellulase *T. reesei* strains as well as provides insight into the regulatory mechanisms of action of TFs for cellulase production.

## Results and discussion

### Construction of transformants with ETAs

**Fig. 1:**
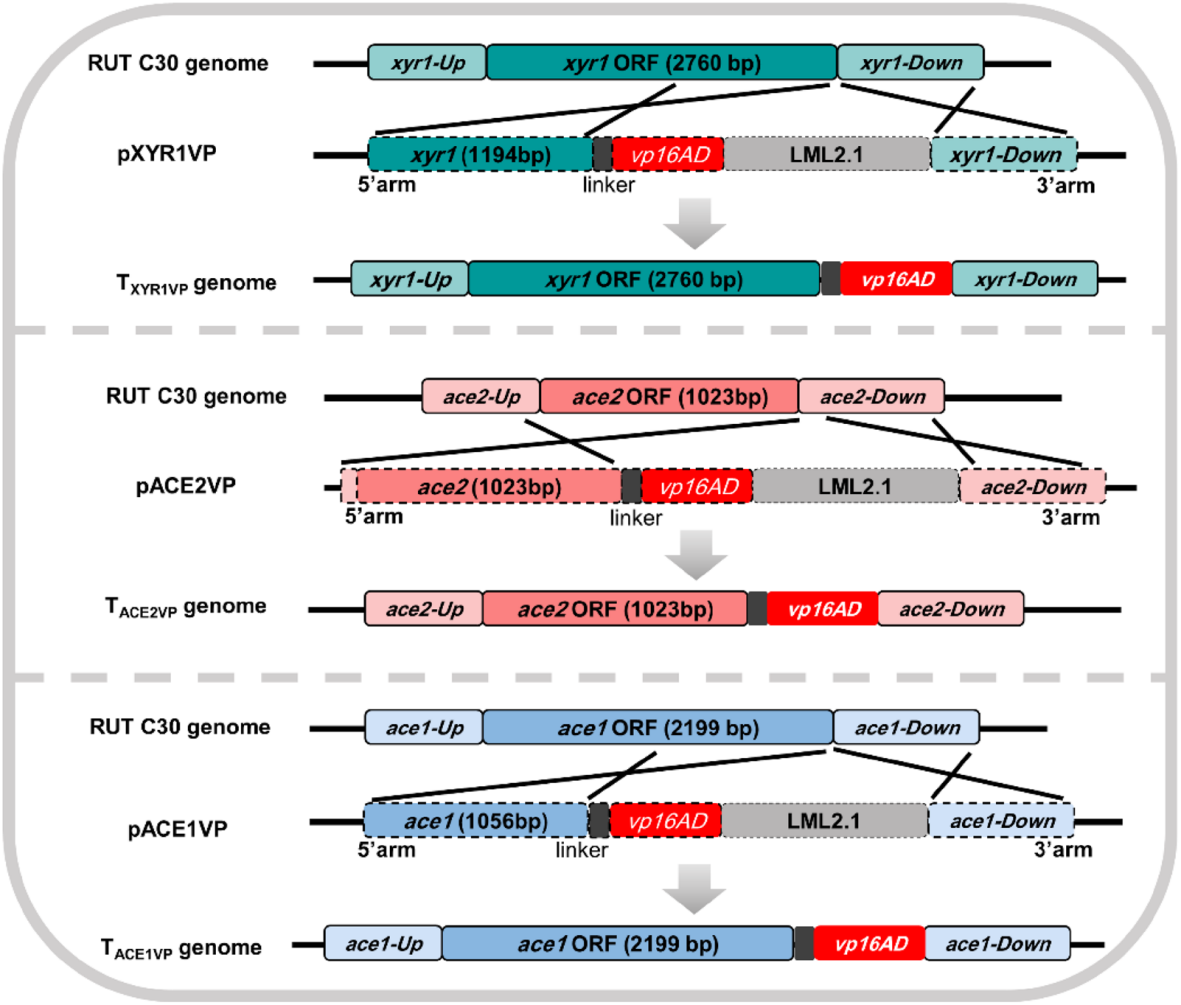
Construction and transfection of the ETAs. A short flexible linker (GGGGSGGGGS) and activation domain of VP16 (vp16AD) were fused with the C terminus of the natural proteins, ACE2 (aa 1–341), ACE1 (aa 1–733), or XYR1 (aa 1–920). The C-terminal last encoding codon of each natural protein was fused to the linker and then fused to the activation domain of VP16 by Seamless Cloning and Assembly Kit (TransGen, Beijing, China). ETAs were used to replace the natural regulators in *T. reesei* RUT C30. Transformants T_ACE2VP_, T_ACE1VP_, and T_XYR1VP_ were obtained after xylose-induced marker rescue

Enhancing the function of TFs to improve cellulase production in *T. reesei* is considered an effective strategy (Zhang et al. 2018). We hypothesized that cellulase production would be increased by fusing the strong transcriptional activation domain of VP16 to the C terminus of a natural TF to enhance its transcriptional activation. Three ETAs including XYR1VP, ACE2VP, and ACE1VP were designed by fusing the VP16 domain to the C terminus of XYR1, ACE2, and ACE1, respectively (Fig. 1). The expression plasmids pXYR1VP, pACE2VP, and pACE1VP for the three ETAs were constructed by ligating the erasable hygromycin selection marker LML2.1 (Fig. 1), which was eliminated by the product of a chimeric Cre recombinase gene (Zhang et al. 2016b). The expression plasmids were introduced into the genome of the *T. reesei* hypercellulolytic mutant RUT C30 by *Agrobacterium*-mediated transformation to replace natural TFs XYR1, ACE2, and ACE1 by homologous double exchange (Fig. 1), eliminating the risk of random insertion and unpredictable mutagenesis. The transformed strains with confirmed gene replacement were selected to eliminate the hygromycin selectable marker by xylose induction to obtain the final transformants. All the transformants were identified as correctly transformed ETA strains and harbored single-copy DNA integration (Additional file 1: Figure S1). Three randomly screened transformants T_XYR1VP_-1/-2/-3, T_ACE2VP_-1/-2/-3, and T_ACE1VP_-1/-2/-3 for each ETA were analyzed further.

### Growth of the transformants expressing ETAs

**Fig. 2:**
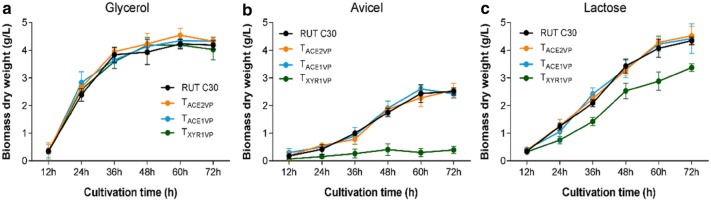
Cell growth differences among T_XYR1VP_, T_ACE2VP_, and T_ACE1VP_ cultured on glycerol (**a**), Avicel (**b**), and lactose (**c**). Error bars show the respective standard deviations of three biological replicates

To determine whether the ETAs helped to increase cellulase production, we first examined the growth of the transformants using parental strain RUT C30 as a control. The growth of ETA transformants T_XYR1VP_, T_ACE2VP_, and T_ACE1VP_ was investigated in the minimal medium (MM) containing glycerol, Avicel, or lactose as the sole carbon source (Fig. 2a–c). Regardless of the carbon sources, growth rates of transformants T_ACE2VP_ and T_ACE1VP_ were not significantly different from that of the control (Fig. 2a–c). Thus, ACE2VP and ACE1VP had no effect on basic cellular metabolism. Similarly, the growth of T_XYR1VP_ showed no significant differences from the control when cultured in glycerol (Fig. 2a). Nevertheless, the growth rates of T_XYR1VP_ on lactose and Avicel were much lower than the growth rate of the control, with growth on Avicel being particularly slow (Fig. 2b, c), indicating that XYR1VP limits cellulase synthesis in T_XYR1VP_.

### XYR1VP abrogated cellulase production

**Fig. 3:**
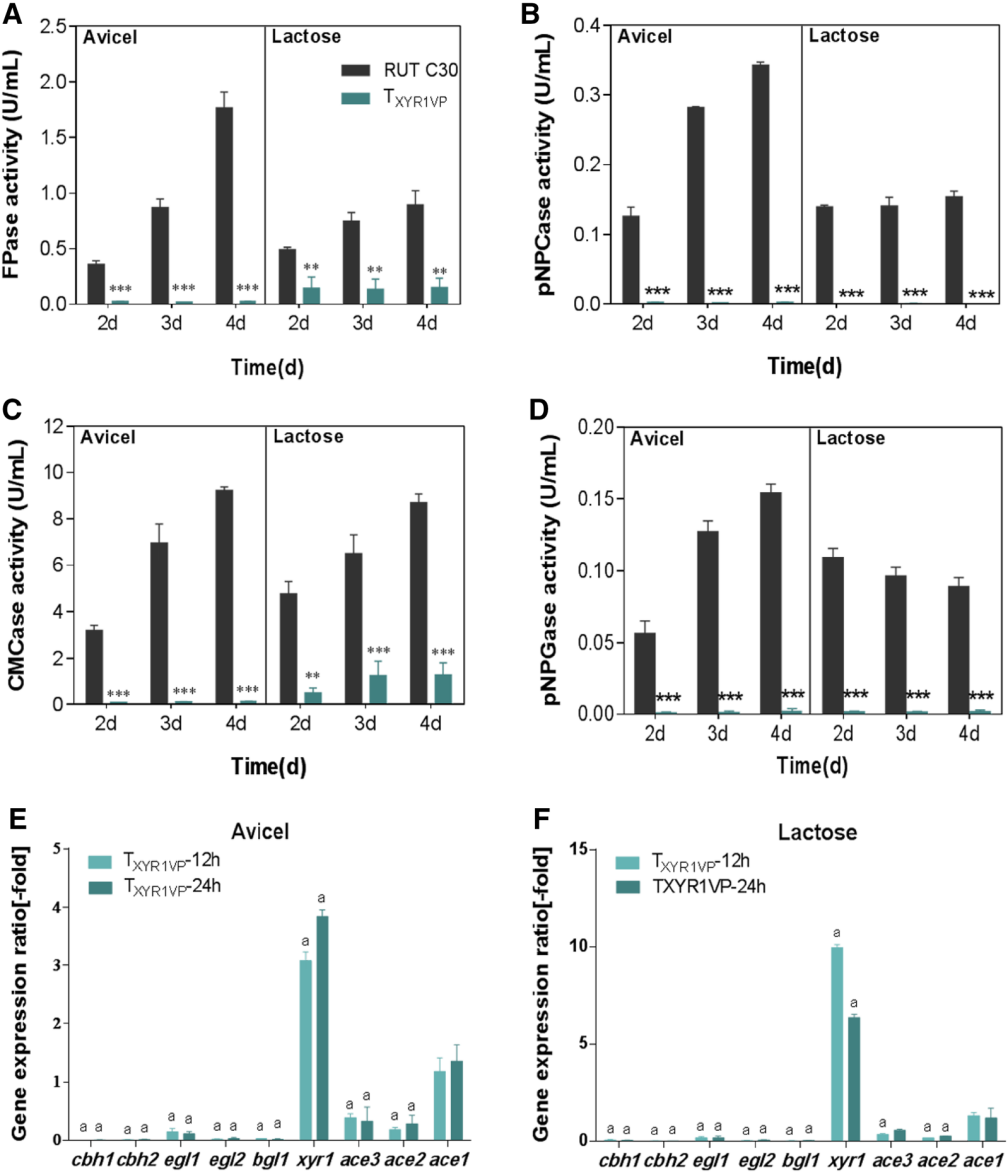
Cellulase production and a comparison of transcript levels of cellulase-related genes between strains T_XYR1VP_ and RUT C30. FPase (**A**), *p*NPCase (**B**), CMCase (**C**), and *p*NPGase (**D**) activities of RUT C30 and T_XYR1VP_ after a switch from glycerol to 20 g/L Avicel or lactose. Enzymatic activities were measured at 2, 3, and 4 days after the transfer. Error bars show the respective standard deviations of three biological replicates; asterisks indicate significant differences (*P ≤ 0.05; **P ≤ 0.01; ***P ≤ 0.001; *n.s.* not significant) between the transformants and RUT C30, as assessed by Student’s *t* test. Expression ratios of cellulase-related genes on 20 g/L Avicel (**E**) or lactose (**F**) for 12 and 24 h after the switch from glycerol. Data on T_XYR1VP_ transformants were normalized to the corresponding gene expression levels at the same time points in RUT C30. Values represent the mean of three biological replicates, and the error bars denote standard deviations. Gene expression ratios greater than twofold or less than 0.5-fold are marked with “a”

To test whether XYR1VP decreases cellulase production, we determined the cellulase-related activities of T_XYR1VP_ under Avicel and lactose culture conditions. T_XYR1VP_ manifested nearly no filter paper activity (FPA) in the Avicel- or lactose-based medium (Fig. 3A). Similarly, the *p*-nitrophenol-d-cellobioside hydrolase (*p*NPCase), the sodium salt of carboxymethyl cellulose hydrolase (CMCase), and the 4-nitrophenyl-beta-d-galactopyranoside hydrolase (*p*NPGase) activities in T_XYR1VP_ were nearly absent as compared to the control (Fig. 3B–D). Additionally, the transcript levels of cellulase-related genes *cbh1*, *cbh2*, *egl1*, and *egl2* were low compared to those in the control (Fig. 3E, F), suggesting that XYR1VP abrogated cellulase production. Consequently, the dramatically decreased growth rate of T_XYR1VP_ on Avicel resulted from its cellulase-negative phenotype in the presence of inducing carbon sources (Avicel and lactose). A lack of cellulase hindered the growth of T_XYR1VP_ in the Avicel-based medium. The cellulase-free phenotype was observed not only in XYR1VP transformants but also in ACE3VP transformants, in which the strong transcriptional activation domain of VP16 was fused to the C terminus of ACE3 (data not shown).

Of note, although cellulase production of T_XYR1VP_ in lactose- and Avicel-based media was abrogated, xylanase production of T_XYR1VP_ was unrepressed and even enhanced (Additional file 1: Figure S2a, b). T_XYR1VP_ showed a xylanase upregulation phenotype in glycerol- and lactose-based media, and a similar xylanase activity (U/mL) was observed in the Avicel-based medium as compared to that in the control. Additionally, T_XYR1VP_ showed higher xylanase activities per unit of biomass with a 51% increase in xylanase I activity and an 80% increase in xylanase II activity compared to that in RUT C30 (Additional file 1: Figure S2a, b). Elevated transcript levels of xylanase genes were detected in T_XYR1VP_ regardless of the carbon sources; this finding is consistent with the enhanced xylanase activities (Additional file 1: Figure S2c–e). T_XYR1VP_, a xylanase-hyperproducing strain with a cellulase-free phenotype, can be employed in some industries where xylanase is desired, while cellulase is undesirable, e.g., in paper recycling (Buchert et al. 1998).

The transcript levels of TFs *xyr1*, *ace3*, *ace2*, and *ace1* in T_XYR1VP_ were also analyzed. As shown in Fig. 3E, F, *ace3* and *ace2* transcript levels were lower in T_XYR1VP_ than in the control (the gene expression ratio was less than 0.5-fold). The transcript levels of the x*yr1* domain were higher than those of the control (the expression ratio was more than threefold) and the transcript levels of *ace1* had no significant differences from those of RUT C30, which cannot explain the cellulase-negative and xylanase overexpression phenotypes of T_XYR1VP_.

XYR1VP abolished cellulase production and increased xylanase production in T_XYR1VP_. By contrast, overexpression of *xyr1* has been found not only to markedly enhance xylanase activity but also to increase cellulase activity in transformants (Mach-Aigner et al. 2008; Uzbas et al. 2012). Therefore, we hypothesized that the C-terminal fusion of XYR1 would alter the function of XYR1VP. Using COILS (Lupas et al. 1991; Lichius et al. 2015) predicted that the C terminus of XYR1 is a coiled-coil domain and likely mediates homodimerization of XYR1. Therefore, fusing the VP16 activation domain at the C terminus of XYR1 abrogated the expression of cellulase genes in *T. reesei*. It is very important to study the function of the C terminus of XYR1. Nonetheless, the positive effect on xylanase gene expression observed after fusion of the VP16 activation domain indicated distinct regulatory mechanisms between cellulase and xylanase genes in terms of transcription. The different responsiveness of XYR1 to cellulase and xylanase was also observed by Pucher et al. (2011). Notably, the cellulolytic regulon of XYR1 is positively affected, whereas the xylanolytic regulon is affected negatively in a *T. reesei* QM9414 strain constitutively expressing *xyr1* (Pucher et al. 2011). These different regulatory mechanisms for cellulase gene transcription are supported by in silico identification of XYR1-binding sites in promoter regions, which are reported to occur at dramatically different frequencies in the promoter region of *cbh1* and *xyn1*: 14-fold for *cbh1* and only 4-fold for *xyn1* (Rauscher et al. 2006; Furukawa et al. 2009).

### ACE2VP enhanced cellulase production

**Fig. 4:**
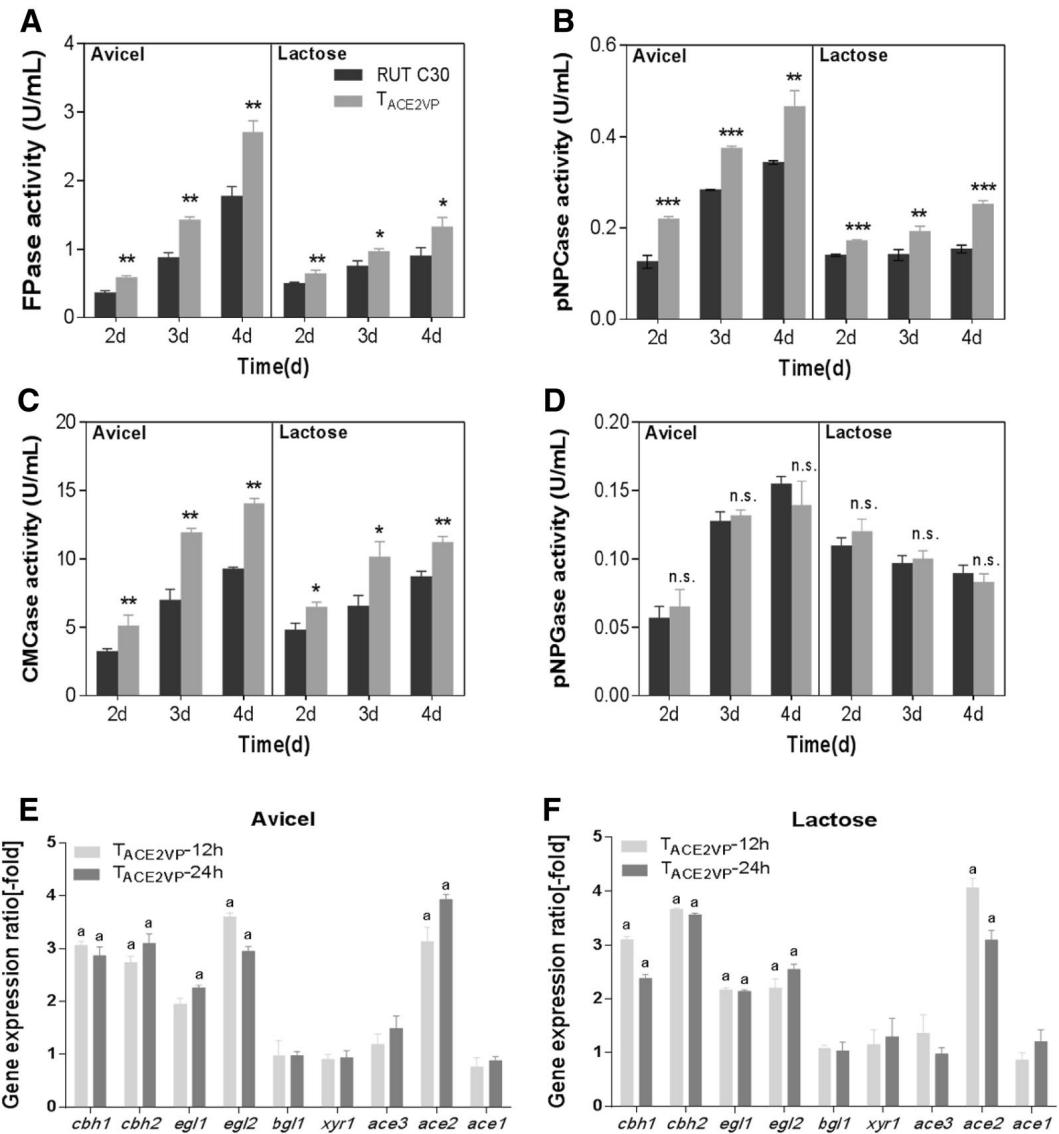
Cellulase production and a comparison of transcript levels of cellulase-related genes between T_ACE2VP_ and RUT C30. FPase (**A**), *p*NPCase (**B**), CMCase (**C**), and *p*NPGase (**D**) activities of RUT C30 and T_ACE2VP_ after the switch from glycerol to 20 g/L Avicel or lactose. Enzymatic activities were measured at 2, 3, and 4 days after the transfer. Error bars show the respective standard deviations of three biological replicates; asterisks indicate significant differences (*P ≤ 0.05; **P ≤ 0.01; ***P ≤ 0.001; *n.s.* not significant) between the transformants and RUT C30, as assessed by Student’s *t* test. Expression ratios of cellulase-related genes on 20 g/L Avicel (**E**) or lactose (**F**) for 12 and 24 h after the switch from glycerol. Data on T_ACE2VP_ transformants were normalized to the corresponding gene expression at the same time points in RUT C30. Values represent the mean of three biological replicates, and error bars denote standard deviations. Gene expression ratios greater than twofold or less than 0.5-fold are marked with “a”

The cellulase-related activities of ACE2VP transformant T_ACE2VP_ were detected to verify the effect of ACE2VP on cellulase production under Avicel and lactose culture conditions. T_ACE2VP_ had a significantly improved FPase activity (Fig. 4A), *p*NPCase activity (Fig. 4B), and CMCase activity (Fig. 4C) in both Avicel- and lactose-based media. The highest FPase activity of T_ACE2VP_ was 2.7 ± 0.2 U/mL in the Avicel-based medium on day 4; this effect was stronger by approximately 52% as compared to the control (Fig. 4A). In the lactose-based medium, FPase activity also increased by approximately 48% on day 4 as compared to the control (Fig. 4A). Additionally, the amount of protein secreted from T_ACE2VP_ increased (Additional file 1: Figure S3a). Consistent with the improved cellulase production, the transcript levels of *cbh1*, *cbh2*, *egl1*, and *egl2* in T_ACE2VP_ significantly increased by 2.0- to 3.7-fold in lactose- and Avicel-based media for 12 and 24 h subculturing (Fig. 4D, E). It has been reported that deleting *ace2* reduces total cellulase activity by 30–70% on cellulose (Aro et al. 2001). Because ACE2 was replaced by an ETA (ACE2VP), cellulase production was enhanced.

Nonetheless, there were no significant differences in the *p*NPGase and xylanase activities between T_ACE2VP_ and control in either Avicel- or lactose-based media (Fig. 4D; Additional file 1: Figure S3b, c). Moreover, the transcript levels of *bgl1*, *xyn1*, and *xyn2* in T_ACE2VP_ showed no significant differences in the lactose- or Avicel-based medium as compared to the control (Fig. 4D, E; Additional file 1: Figure S3d, e). One study revealed no effect on *xyn1* expression in strains in which *ace2* was deleted (Aro et al. 2001). It is possible that xylanase production was not improved in T_ACE2VP_ because ACE2 did not affect *xyn1* expression, as was the case for ACE2VP. ACE2 can affect the expression of *xyn2* and *bgl1* (Aro et al. 2001; Stricker et al. 2008). Additionally, phosphorylation and dimerization are prerequisites for ACE2 to bind the promoters of target genes (Stricker et al. 2008). Fusion of the VP16 domain to the C terminus of ACE2 may influence the native effects on *xyn2* and *bgl1* expression.

We also analyzed the relative transcript levels of TFs *xyr1*, *ace3*, *ace2* (*ace2vp* for T_ACE2VP_), and *ace1* in T_ACE2VP_. The transcript level of *ace2vp* in T_ACE2VP_ was approximately 4.0-fold higher than that of *ace2* in the control (Fig. 4D, E), indicating that the VP16 domain fused at the C terminus of ACE2 elevated its own transcript level. The increased transcript levels of *ace2vp* in T_ACE2VP_ may have also promoted cellulase production. The transcript levels of *xyr1*, *ace3*, and *ace1* in T_ACE2VP_ were similar to those in the control (an expression increase was approximately onefold), indicating that ACE2VP mainly affects the expression of cellulase genes and its own expression and has little effect on other TFs.

### ACE1VP enhanced cellulase production

**Fig. 5:**
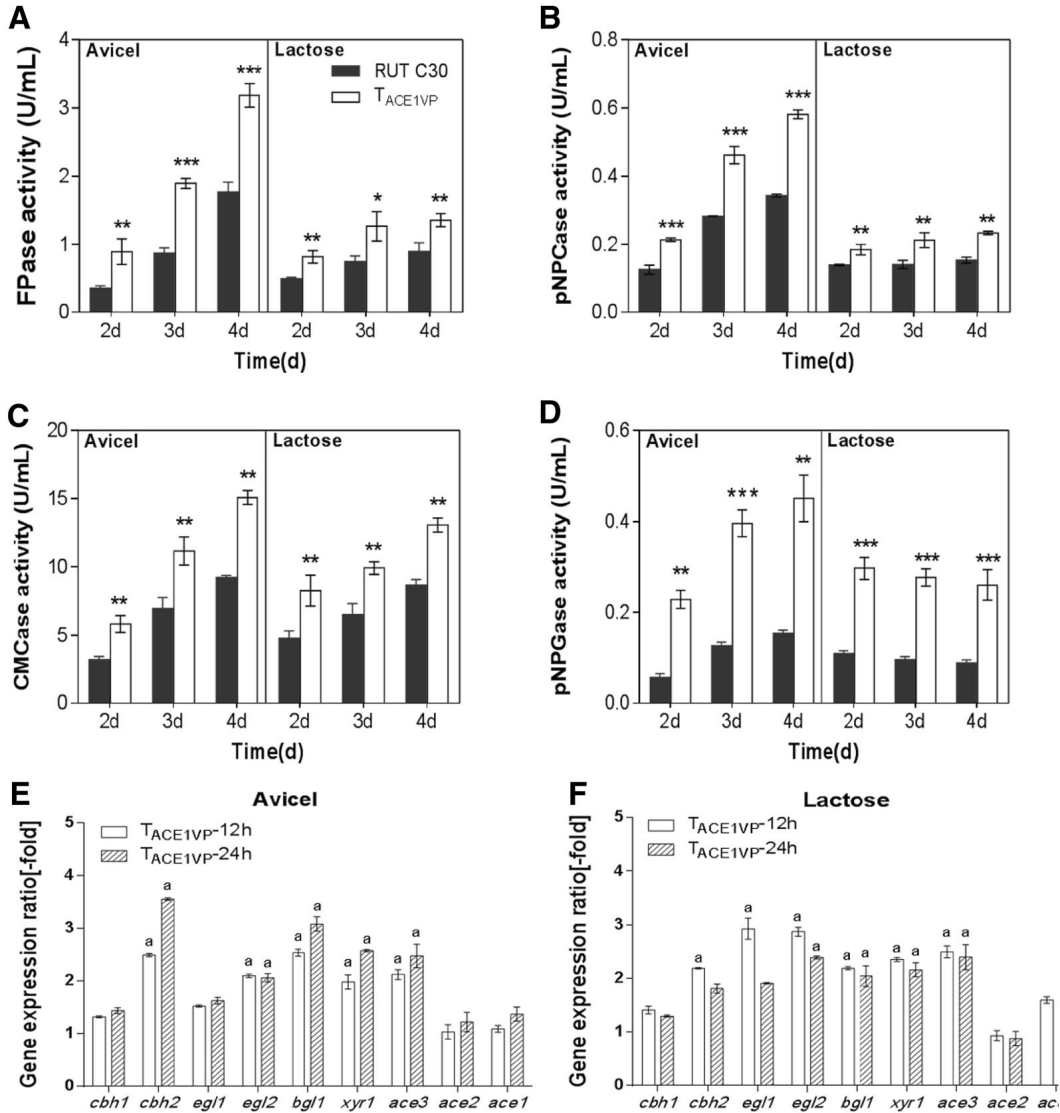
Cellulase production and a comparison of transcript levels of cellulase-related genes between T_ACE1VP_ and RUT C30. FPase (**A**), *p*NPCase (**B**), CMCase (**C**), and *p*NPGase (**D**) activities of RUT C30 and T_ACE1VP_ after the switch from glycerol to 20 g/L Avicel or lactose. Enzymatic activities were measured at 2, 3, and 4 days after the transfer. Error bars show the respective standard deviations of three biological replicates; asterisks indicate significant differences (*P ≤ 0.05; **P ≤ 0.01; ***P ≤ 0.001; *n.s.* not significant) between the transformants and RUT C30, as assessed by Student’s *t* test. Expression ratios of cellulase-related genes on 20 g/L Avicel (**E**) or lactose (**F**) for 12 and 24 h after the switch from glycerol. The data on T_XYR1VP_ transformants were normalized to the corresponding gene expression at the same time points in RUT C30. Values represent the mean of three biological replicates, and the error bars denote standard deviation. Gene expression ratios greater than twofold or less than 0.5-fold are marked with “a”

To investigate the effect of ACE1VP on cellulase production, on the cellulase-related activities, and on the amount of protein secreted by ACE1VP transformants, T_ACE1VP_ and RUT C30 were examined under Avicel and lactose culture conditions. The amount of protein secreted from T_ACE1VP_ notably increased (Additional file 1: Figure S4a). Additionally, elevated cellulase activities were observed in T_ACE1VP_ as compared to those in the control in both Avicel- and lactose-based media (Fig. 5A–D). Cellulase production by T_ACE1VP_ was higher in the Avicel-based medium than in the lactose-based medium. The highest FPase activity of T_ACE1VP_ transformants was 3.2 ± 0.2 U/mL in the Avicel-based medium, with an increase in activity of approximately 80% on day 4 compared to that in the control; this result is consistent with the enhanced *p*NPCase, CMCase, and *p*NPGase activities (Fig. 5B–D). Xylanase activities (on day 4) of T_ACE1VP_ transformants rose by nearly 50% in the Avicel-based medium as compared to the control (Additional file 1: Figure S4b, c). These results suggested that ACE1VP promotes cellulase and xylanase production in both Avicel- and lactose-based media. It has been reported that *ace1* is a repressor and deleting *ace1* increases the amounts of cellulase produced in *T. reesei* (Aro et al. 2003). Here, ACE1VP is an artificial TF that promotes cellulase production. ACE1VP did not elevate the transcript level of itself but promoted the transcript levels of the cellulase genes and of transcriptional activators *xyr1* and *ace3*. Therefore, ACE1VP harbors positive transcriptional effects for cellulase production, whereas native ACE1 had a negative effect on cellulase production.

The transcript levels of *cbh1*, *cbh2*, *egl1*, *egl2*, *bgl1*, *xyn1*, and *xyn2* in T_ACE1VP_ increased 1.3- to 3.8-fold in the Avicel-based medium as compared to the control for 12 and 24 h subculturing (Fig. 5E; Additional file 1: Figure S4d, e). Similarly, there was a remarkable increase (~ 1.3 to 3.0-fold) in the transcript levels of cellulase genes in T_ACE1VP_ as compared to the control strain in the lactose-based medium (Fig. 5F). The elevated transcript levels were consistent with increased enzyme production by T_ACE1VP_ in both Avicel- and lactose-based media. Aro et al. (2003) reported that deleting *ace1* increased the *cbh1*, *cbh2*, *egl1*, *egl2*, *xyn1*, and *xyn2* transcription levels in the Avicel-based medium. Our results revealed that ACE1VP acts as a positive regulator improving cellulase and xylanase production in Avicel and lactose media.

The transcript levels of *ace2* and *ace1* (*ace1vp* for T_ACE1VP_) in T_ACE1VP_ were similar to those in the control (the expression ratio was approximately onefold), whereas the transcript levels of *xyr1* and *ace3* in T_ACE1VP_ obviously increased in both Avicel-and lactose-based media as compared to the control (Fig. 5E, F). The *xyr1* and *ace3* expression ratios were approximately 1.9- to 2.6-fold in the Avicel- and lactose-based medium (Fig. 5E, F), indicating that the VP16 domain fused to the C terminus of ACE1 raised the transcript levels of key transcriptional activators XYR1 and ACE3. The increased transcript levels of *xyr1* and *ace3* in T_ACE1VP_ reflect improved cellulase and xylanase production; this result is consistent with other reports of overexpression of *xyr1* and *ace3* in *T. reesei* (Mach-Aigner et al. 2008; Uzbas et al. 2012; Häkkinen et al. 2014).

### Hydrolysis of corn stover by cellulase from T_ACE1VP_ and T_ACE2VP_

**Fig. 6:**
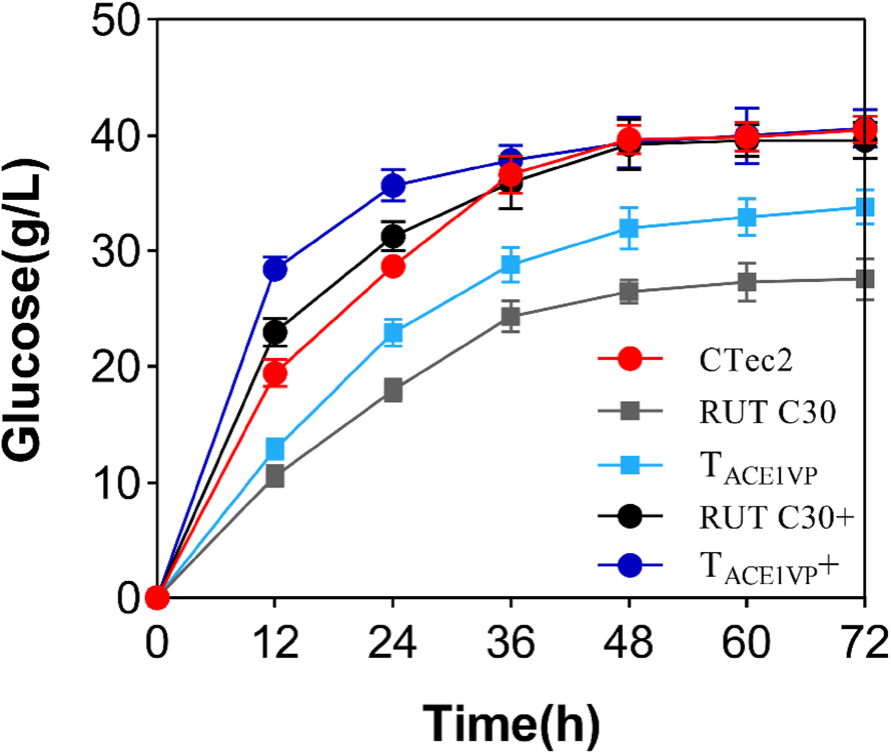
Hydrolysis of corn stover by CTec2 and by the crude enzyme from T_ACE1VP_ or RUT C30. The crude enzymes from T_ACE1VP_ or RUT C30 were either supplemented with β-glucosidase (Sunson Enzymes) (T_ACE1VP_+, RUT C30+) or not supplemented with β-glucosidase (T_ACE1VP_, RUT C30) at the CBU/FPA ratio of 2. Commercial cellulase CTec2 (Novozymes, Bagsvaerd, Denmark) served as the control. Enzymatic hydrolysis was performed at 15 FPA/(g pretreated corn stover). Values represent the mean and standard deviation of triplicate measurements

T_ACE1VP_ and T_ACE2VP_ outperformed the control by showing markedly increased cellulase production. The crude cellulase produced by T_ACE1VP_ and T_ACE2VP_ was used to hydrolyze pretreated and biodetoxified corn stover (Qiu et al. 2017), with strain RUT C30 serving as a control. At the same FPase loading (15 U/g pretreated corn stover), 33.8 ± 1.5 g/L glucose was produced after 72 h of incubation by crude cellulase from T_ACE1VP_. This result was approximately 22.6% higher than that produced by RUT C30 (Fig. 6). The increased glucose release resulted from enhanced *p*NPGase activity in T_ACE1VP_, which can hydrolyze cellobiose to glucose. There was no significant difference in the amount of glucose released between the crude enzymes from T_ACE2VP_ and the control (Additional file 1: Figure S5); these data are in agreement with the unimproved level of *p*NPGase activity from T_ACE2VP_ in comparison with the control. Nonetheless, the production of cellulase by T_ACE2VP_ was improved, resulting in a smaller crude cellulase volume loading as compared to the control. Thus, the costs of cellulase from T_ACE1VP_ to T_ACE2VP_ were reduced successfully.

After the supplementation with commercial β-glucosidase (4000 CBU/mL, Sunson Enzymes, Beijing, China) at the 2:1 CBU/FPA ratio, the glucose release from T_ACE1VP_ (40.6 ± 1.6 g/L) and T_ACE2VP_ (39.4 ± 2.1 g/L) was nearly the same as that observed with the commercial enzyme CTec2 (40.5 ± 1.2 g/L) (Fig. 6) at 72 h, revealing that the enzymes produced by T_ACE1VP_ and T_ACE2VP_ effectively hydrolyzed the pretreated corn stover. Additionally, cellulase from strain RUT C30 supplemented with β-glucosidase showed similar hydrolysis performance relative to the commercial CTec2. This phenomenon has been described in other reports too (Zhang et al. 2017, 2018), suggesting that the cellulase secreted by strain RUT C30 contains insufficient amounts of β-glucosidase (Pryor and Nahar 2015; Li et al. 2016; Zhang et al. 2016a). The T_ACE1VP_ strain showed higher β-glucosidase activity, which might be the reason for the improved hydrolysis yield with T_ACE1VP_.

## Conclusion

We designed a universal and simple pattern strategy for enhancing the transcriptional activation of native regulators ACE2, ACE1, and XYR1 from *T. reesei* by linking their C terminus with the strong transcriptional activation domain of VP16. XYR1VP significantly improved xylanase gene transcription, while it abrogated cellulase gene expression. ACE2VP improved cellulase production in both lactose and Avicel media but was ineffective at inducing *p*NPGase and xylanase production. Moreover, ACE1VP functioned as a positive regulator of cellulase and xylanase expression. This is the first study to report the effects of ETAs on cellulase and xylanase production; these data are helpful for strain improvement of *T. reesei*. Additionally, our universal and simple pattern strategy for constructing ETAs can serve as an alternative genetic engineering method for increasing the yield of industrial products in other hosts.

## Methods

### Strains and media

Hypercellulolytic strain *T. reesei* RUT C30 (ATCC 56765) serving as a parental strain and control was purchased from ATCC (Manassas, VA, USA). *Escherichia coli* DH5α and *Agrobacterium tumefaciens* AGL-1 were used as host strains for recombinant DNA manipulations and for *Agrobacterium*-mediated transformation (Michielse et al. 2008). DH5α and AGL-1 cells were cultured in the Luria–Bertani medium.

### Construction of ETAs and their expression plasmids

Three ETAs—XYR1VP, ACE2VP, and ACE1VP—were designed by fusing the VP16 domain to the C terminus of XYR1, ACE2, or ACE1, respectively (Fig. 1). The C-terminal coding sequences and downstream sequences of natural TFs (ACE2, ACE1, and XYR1) were employed as the left (5′-) and right (3′-) homologous arms, respectively (Fig. 1). The linker and activation domain of VP16 of the herpes simplex virus protein were amplified by PCR from pG1V (Wang et al. 2014). All the primers are listed in Table S1 (Additional file 1). An unmarked genetic modification tool LML2.1 (Zhang et al. 2016b) served as the skeleton of all the plasmids, in which the hygromycin resistance gene was removed by xylose-induced Cre recombinase. The amplified 5′- and 3′-fragments were fused to the corresponding sites *Pac*I/*Xba*I and *Swa*I of LML2.1, respectively, with the Seamless Cloning and Assembly Kit (TransGen, Beijing, China). The resulting vectors were named as pXYR1VP, pACE1VP, and pACE2VP (Fig. 1).

### Transformation of *T. reesei* and verification of the transformed clones

*Agrobacterium*-mediated transformation (Michielse et al. 2008), transformed-clone verification, and xylose-induced marker rescue (Zhang et al. 2016b) were performed to obtain the ETA-transformed strains T_XYR1VP_, T_ACE1VP_, and T_ACE2VP_. Single-copy DNA integration in the transformed clones was verified by diagnostic PCR, amplicon sequencing, and quantitative PCR (qPCR) as described by Li et al. (2017). The related primers are presented in Additional file 1: Figure S1 and Table S1. TransStart TipTop Green qPCR SuperMix (TransGen) was used for qPCR assays. For each ETA, three randomly selected transformants (T_XYR1VP_-1/-2/-3, T_ACE2VP_-1/-2/-3, and T_ACE1VP_-1/-2/-3) were collected.

### The biomass concentration assay

For the fungal growth assay, conidia (final concentration 10^6^/mL) from each *T. reesei* strain were inoculated into 100 mL of the minimal medium [MM, (NH_4_)_2_SO_4_ 5 g/L; Urea 0.3 g/L; KH_2_PO_4_ 15 g/L; CaCl_2_ 0.6 g/L; MgSO_4_ 0.6 g/L; FeSO_4_·7H_2_O 5 mg/L; ZnSO_4_·7H_2_O 1.4 mg/L; CoCl_2_·6H_2_O 2 mg/L; pH 5.5] containing 20 g/L glycerol, lactose, or Avicel in 500 mL Erlenmeyer flasks and were cultivated by shaking (200 rpm) at 28 °C for 72 h. Two milliliters of the culture liquid was collected every 12 h for biomass concentration analysis as described by Bischof et al. (2013). Intracellular protein contents were measured by means of the Modified Lowry Protein Assay Kit (Sangon Biotech, Shanghai, China). The biomass (in dry weight per liter) was quantified by calculating the intracellular protein content in a glycerol-, Avicel-, or lactose-based medium assuming 0.32 g of intracellular protein per gram of dry biomass (Bischof et al. 2013).

### Enzyme production in a flask

For the cellulase production assay, conidia (10^7^/mL) from each *T. reesei* strain were inoculated into 100 mL of the Mandels Andreotti (MA) medium (Mandels and Andreotti 1978) supplemented with 1 g/L peptone (Oxoid, Basingstoke, England) and 20 g/L glycerol in 500 mL Erlenmeyer flasks and were cultivated by shaking (200 rpm) at 28 °C for 2 days. Pregrown mycelia were harvested by filtration, washed with distilled water, and dried with sterile filter paper. Equal amounts of these mycelia were transferred into two 50 mL aliquots of fresh MA supplemented with 1 g/L peptone with 20 g/L lactose, or Avicel as the sole carbon source (Chen et al. 2016). Incubation was continued at 28 °C with shaking at 200 rpm for 4 days. When enzyme production was analyzed in glycerol, the mycelial culture time was prolonged to 4 days without transfer. Two milliliters of the culture liquid was collected via centrifugation at 14,000×*g* and 4 °C for 10 min. The culture supernatants were subjected to cellulase activity measurements. The mycelia were washed with distilled water, dried with sterile filter paper, and subjected to RNA extraction and biomass concentration assays.

### RNA extraction and Real-time quantitative PCR (RT-qPCR) analysis

RNA was extracted using the FastRNA Pro Red Kit (MP Biomedicals, Santa Ana, CA, USA). cDNA was synthesized with TransScript All-in-One First-Strand cDNA Synthesis SuperMix for qPCR (TransGen). The levels of gene-specific mRNA were assessed by RT-qPCR on an ABI StepOne Plus thermocycler (Applied Biosystems, Foster City, CA, USA). The primers are described in Additional file 1: Table S1. The cycling conditions comprised 30 s initial denaturation and polymerase activation at 95 °C, followed by 40 cycles of 5 s at 95 °C and 60 s at 64 °C. Threshold cycle (Ct) values and PCR efficiency rates were used to calculate relative expression quantities by the ABI software. Transcript levels of target genes were normalized to *sar1* expression (Steiger et al. 2010) by the 2^−ΔΔCt^ method.

### Enzymatic activity and the protein concentration assay

Produced cellulase activities against filter paper (FP), *p*-nitrophenyl-d-cellobioside (*p*NPC), sodium salt of carboxymethyl cellulose (CMC), and 4-nitrophenyl-beta-d-galactopyranoside (*p*NPG) were measured at pH 5.0 throughout cultivation. One unit of FPase or CMCase activity forms 1 μmol of reducing sugar per minute during the hydrolysis reaction, which was quantified by the 3,5-dinitrosalicylic acid method with glucose as a standard (Miller 1959). One unit of *p*NPCase or *p*NPGase activity was defined as the amount of the enzyme needed to produce 1 μmol of *p*-nitrophenol per minute during the hydrolysis reaction. Xylanase I and xylanase II activities were measured by xylan degradation at pH 3.7 and 5.0, respectively, as described by Stricker et al. (2008). One unit of xylanase activity was defined as the amount of the enzyme needed to generate 1 μmol of xylose reducing sugar equivalents per minute under the defined assay conditions. Protein concentration was determined by means of the Modified Lowry Protein Assay Kit (Sangon Biotech, Shanghai, China).

### Enzymatic hydrolysis of pretreated corn stover

Pretreated and biodetoxified corn stover was found to contain 37.6% of cellulose and 4.4% of hemicellulose by dry mass and was kindly provided by Professor Jie Bao (Qiu et al. 2017). The crude enzymes produced by the *T. reesei* ETA transformants and RUT C30 were collected in the Avicel-based MA medium after 4 days of fermentation after the switch from the glycerin-based medium. Saccharification was performed on 10% (w/v) pretreated corn stover as a substrate in a flask with FPase loading (15 U/g dry biomass) at 50 °C and pH 5.0 (50 mM sodium citrate buffer) for 72 h. The enzymes were supplemented with β-glucosidase (Sunson Enzymes) at the CBU/FPA ratio of two to hydrolyze pretreated corn stover when necessary. The glucose release was evaluated as described by Li et al. (2016).

### Statistical analysis

All the experiments were conducted with three biological replicates and three technical replicates for each biological replicate. Student’s two-tailed *t* test was performed in Microsoft Excel (Office 2013) (Microsoft, Redmond, WA, USA) to detect significant differences between two samples. *P* ≤ 0.05 was considered to indicate statistical significance.

## Additional file

**Additional file 1: Table S1.** Primers used in this study. **Figure S1.** A schematic for identification of gene integration and single-copy DNA integration into the genome of transformants. **Figure S2.** Xylanase production and a comparison of transcript levels of xylanase-related genes between T_XYR1VP_ and RUT C30. **Figure S3.** The amount of secreted protein and xylanase production in T_ACE2VP_ and RUT C30. **Figure S4.** The amount of secreted protein and xylanase production in T_ACE1VP_ and RUT C30. **Figure S5.** Hydrolysis of corn stover by CTec2 and by the crude enzyme from T_ACE1VP_ or T_ACE2VP_.

## Abbreviations

ETA: enhanced transcriptional activator
TF: transcription factor
aa: amino acid residues
FPA or FPase: filter paper activity
*p*NPCase: *p*-nitrophenol-d-cellobioside hydrolase activity
CMCase: sodium salt of carboxymethyl cellulose hydrolase activity
*p*NPGase: 4-nitrophenyl-beta-d-galactopyranoside hydrolase activity
*p*NPC: *p*-nitrophenyl-d-cellobioside
*p*NPG: 4-nitrophenyl-beta-d-galactopyranoside
CMC: sodium salt of carboxymethyl cellulose
U: international units
CBU: cellobiase units
RT-qPCR: real-time quantitative polymerase chain reaction
MA medium: Mandels Andreotti medium
MM: minimal medium
