# Supplementary material for: Construction of enhanced transcriptional activators for improving cellulase production in Trichoderma reesei RUT C30
Source: Bioresour Bioprocess. 2018 Aug 18;5(1):40. doi: 10.1186/s40643-018-0226-4 (PMC7101855; doi:10.1186/s40643-018-0226-4)
Supplement: Supplementary file 1 — Additional file 1: Table S1. Primers used in this study. Figure S1. A schematic for identification of gene integration and single-copy DNA integration into the genome of transformants. Figure S2. Xylanase production and a comparison of transcript levels of xylanase-related genes between TXYR1VP and RUT C30. Figure S3. The amount of secreted protein and xylanase production in TACE2VP and RUT C30. Figure S4. The amount of secreted protein and xylanase production in TACE1VP and RUT C30. Figure S5. Hydrolysis of corn stover by CTec2 and by the crude enzyme from TACE1VP or TACE2VP. [file 40643_2018_226_MOESM1_ESM.docx]

**Construction of** **enhanced transcriptional activators for improving cellulase production in *Trichoderma reesei* RUT C30**

**Jiajia Zhang^1^,** **Chuan Wu^1^, Wei Wang^2^,** **Wei Wang^1^*, Dongzhi Wei^1^***

1. New World Institute of Biotechnology, State key Lab of Bioreactor Engineering, East China University of Science and Technology, Shanghai 200237, China.

2. State key Lab of Bioreactor Engineering, East China University of Science and Technology, Shanghai 200237, China.

E-mail address:

*Correspondence: wadexp@ecust.edu.cn, dzhwei@ecust.edu.cn

Jiajia Zhang: zhangjia1114@163.com

Chuan Wu: 1371727710@qq.com

Wei Wang^2^: weiwang@ecust.edu.cn

Dongzhi Wei: dzhwei@ecust.edu.cn

**Table S1. Primers used in this study.**

| Name | Sequences (5’-3’) | Relevant features |
| --- | --- | --- |
| 5-xyr1-f | GATTACGAATTCTTAATTAATGGATGAGCACGACTTGAACAGAA | 5’ –flanks for *xyr1* |
| 5-xyr1-r | TTCCGCCACCGCCaagcttGAGGGCCAGACCGGTTCCGTTA |  |
| 3-xyr1-f | ACTAGTGAGCTCATTTGGAGGCCACTCAATCGTATG | 3’ –flanks for *xyr1* |
| 3-xyr1-r | AGTGCCAAGCTTATTTGAACCTCTTACTCACATTCACTTG |  |
| 5-ace2-f | GATTACGAATTCTTAATTAACGATGGAGGCGATCACGATAA | 5’ –flanks for *ace2* |
| 5-ace2-r | TTCCGCCACCGCCaagcttCTTCAGCAGTCTGGCACTGA |  |
| 3-ace2-f | ACTAGTGAGCTCATTTTATGGACGGCAACGGTGAGG | 3’ –flanks for *ace2* |
| 3-ace2-r | AGTGCCAAGCTTATTTGGAGAAGCAACACGCATCAATCT |  |
| 5-ace1-f | GATTACGAATTCTTAATTAAATGCGGTCCATGGCCCGCCGGAA | 5’ –flanks for *ace1* |
| 5-ace1-r | TTCCGCCACCGCCaagcttCTCTTGAAACCCCTGGTAGTCGA |  |
| 3-ace1-f | ACTAGTGAGCTCATTTAAAGATTGCGACACATACAATGA | 3’ –flanks for *ace1* |
| 3-ace1-r | AGTGCCAAGCTTATTTCATTAGACCGACCTAATTACTTACT |  |
| VP16-f | aagcttGGCGGTGGCGGAAG | VP16 |
| VP16-r | TTAAGTTAACTCTAGATTAGCCGCCGTACTCGTCGATG |  |
| q-sar1-f | TGGATCGTCAACTGGTTCTACGA | qRT-PCR |
| q-sar1-r | GCATGTGTAGCAACGTGGTCTTT |  |
| q-cbh1-f | CTCCATCTCCGAGGCTCTTACC |  |
| q-cbh1-r | GCAAGTGCCGCCATATCTGTTAT |  |
| q-cbh2-f | GCATATTACGCCTCTGAAGTTAGCA |  |
| q-cbh2-r | GCATAGTTACCGCCATTCTTGTTG |  |
| q-egl1-f | GCAGCCTCACCATGAACCAGTA |  |
| q-egl1-r | CACCGTCAGAGTCCAGGAGATAC |  |
| q-egl2-f | AACTACCGCTGGATGCACGA |  |
| q-egl2-r | TAGTCGACGCCCTCGATGAA |  |
| q-bgl1-f | CAAGTGACTGGTGCCGAGGTA |  |
| q-bgl1-r | CGTTGCTGTTCCGCTCTGAC |  |
| q-xyn1-f | GGTTGGACGACTGGATCT |  |
| q-xyn1-r | GGTTGTCCTCCATGATGTAG |  |
| q-xyn2-f | CATCGTCGAGAACTTTGGCA |  |
| q-xyn2-r | GCGTGCGGTAAATGTCGTAG |  |
| q-xyn3-f | ACTCTGATCTGGCACTCGCA |  |
| q-xyn3-r | ACGTCCCAAGCACGAATCTT |  |
| q-xyn4-f | CAGACTGAGTGGGCAGATGG |  |
| q-xyn4-r | CCTGAGGTGTCGCTGTTGAC |  |
| q-xyn5-f | CTGGTGGATCTGCTCCCATC |  |
| q-xyn5-r | GAGCAGATCCACCAGGGTTC |  |
| q-bxl-f | TACGGCCATGCTGTTTGTTC |  |
| q-bxl-r | AATCCGACGAGCCACTTGTT |  |
| q-xyl-f | GACACCGTGTACAACGCCAT |  |
| q-xyl-r | CATAGTCACAGGCACCGTCAA |  |
| q-ace1-f | GACAAGACGGATGTGTTCCAG |  |
| q-ace1-r | GTTGAAGATGTCGGGCTGTG |  |
| q-ace2-f | TCAACATCCTCCACCACCAGTC |  |
| q-ace2-r | TGTCGGCGTACTCTCTCAGC |  |
| q-xyr1-f | CTTCCTCCTCCTGCTCATCG |  |
| q-xyr1-r | TCGTGTGCCCTAACAATGGTC |  |

**Figure S1. A schematic for identification of gene integration and single-copy DNA integration into the genome of transformants.** Primers showed in red are used to verify the gene integration. Primers showed in black are used to identify the copy number of integrated genes.

**Figure S2. Xylanase production and a comparison of transcript levels of xylanase-related genes between T_XYR1VP_ and RUT C30.**

Xylanase I **(a)** and Xylanase II **(b)** activity from T_XYR1VP_ and RUT C30 and after a switch from glycerol to 20 g/L glycerol, avicel or lactose, with U per ml (left) and U per mg biomass dry weight (right). Enzyme activities were measured at 2, 3, and 4 days after transfer. Error bars show the respective standard deviation of three biological replicates; asterisks indicate significant differences (*P ≤ 0.05; **P ≤ 0.01; ***P ≤ 0.001; n.s.: not significant) between the transformants and RUT C30, as assessed by Student’s *t* test. Xylanase-related genes expression ratios on 20 g/l glycerol **(c)**, Avicel **(d)** or lactose **(e)** for 12 and 24 h after the switch from glycerol. Data on T_XYR1VP_ transformants were normalized to the corresponding gene expression levels at the same time in RUT C30. Values represent the mean of three biological replicates and error bars show the standard deviation. Gene expression ratios greater than 2-fold or less than 0.5-fold are marked with “a”.

**Figure S3. The amount of secreted protein and xylanase production in T_ACE2VP_ and RUT C30.**

The amount of secreted protein **(a)**, Xylanase I **(b)** and Xylanase II **(c)** activity from T_ACE2VP_ and RUT C30 and after a switch from glycerol to 20 g/L Avicel or lactose. Enzyme activities were measured at 2, 3, and 4 days after transfer. Error bars show the respective standard deviation of three biological replicates; asterisks indicate significant differences (*P ≤ 0.05; **P ≤ 0.01; ***P ≤ 0.001; n.s.: not significant) between the transformants and RUT C30, as assessed by Student’s *t* test. The comparison of transcript levels of *xyn1* and *xyn2* on 20 g/L avicel **(d)** or lactose **(e)** for 12 and 24 h after the switch from glycerol. Data on T_ACE2VP_ transformants were normalized to the corresponding gene expression at the same time points in RUT C30. Values represent the mean of three biological replicates and error bars show the standard deviations. Gene expression ratios greater than 2-fold or less than 0.5-fold are marked with “a”.

**Figure S4. The amount of secreted protein and xylanase production in T_ACE1VP_ and RUT C30.**

The amount of secreted protein **(a)**, Xylanase I **(b)** and Xylanase II **(c)** activity from T_ACE1VP_ and RUT C30 and after a switch from glycerol to 20 g/L Avicel or lactose. Enzyme activities were measured at 2, 3, and 4 days after transfer. Error bars show the respective standard deviation of three biological replicates; asterisks indicate significant differences (*P ≤ 0.05; **P ≤ 0.01; ***P ≤ 0.001; n.s.: not significant) between the transformants and RUT C30, as assessed by Student’s *t* test. The comparison of transcript levels of *xyn1* and *xyn2* n 20 g/L Avicel **(d)** or lactose **(e)** for 12 and 24 h after the switch from glycerol. Data on T_ACE1VP_ transformants were normalized to the corresponding gene expression at the same time points in RUT C30. Values represent the mean of three biological replicates and error bars show the standard deviations. Gene expression ratios greater than 2-fold or less than 0.5-fold are marked with “a”.

**Figure S5. Hydrolysis of corn stover by CTec2 and the crude enzyme from T_ACE1VP_ or T_ACE2VP_.**

The crude enzymes from T_ACE1VP_, T_ACE2VP_ or RUT C30 were either supplemented with β-glucosidase (Sunson Enzymes) (T_ACE1VP_+, T_ACE2VP_+, RUT C30+) or without β-glucosidase (T_ACE1VP_, T_ACE2VP_, RUT C30) at the CBU/FPA ratio of 2. Commercial cellulase CTec2 (Novozymes, Bagsvaerd, Denmark) served as the control. Enzymatic hydrolysis was performed at 15 FPA/(g pretreated corn stover). Values represent the mean and standard deviation of triplicate measurements.

**
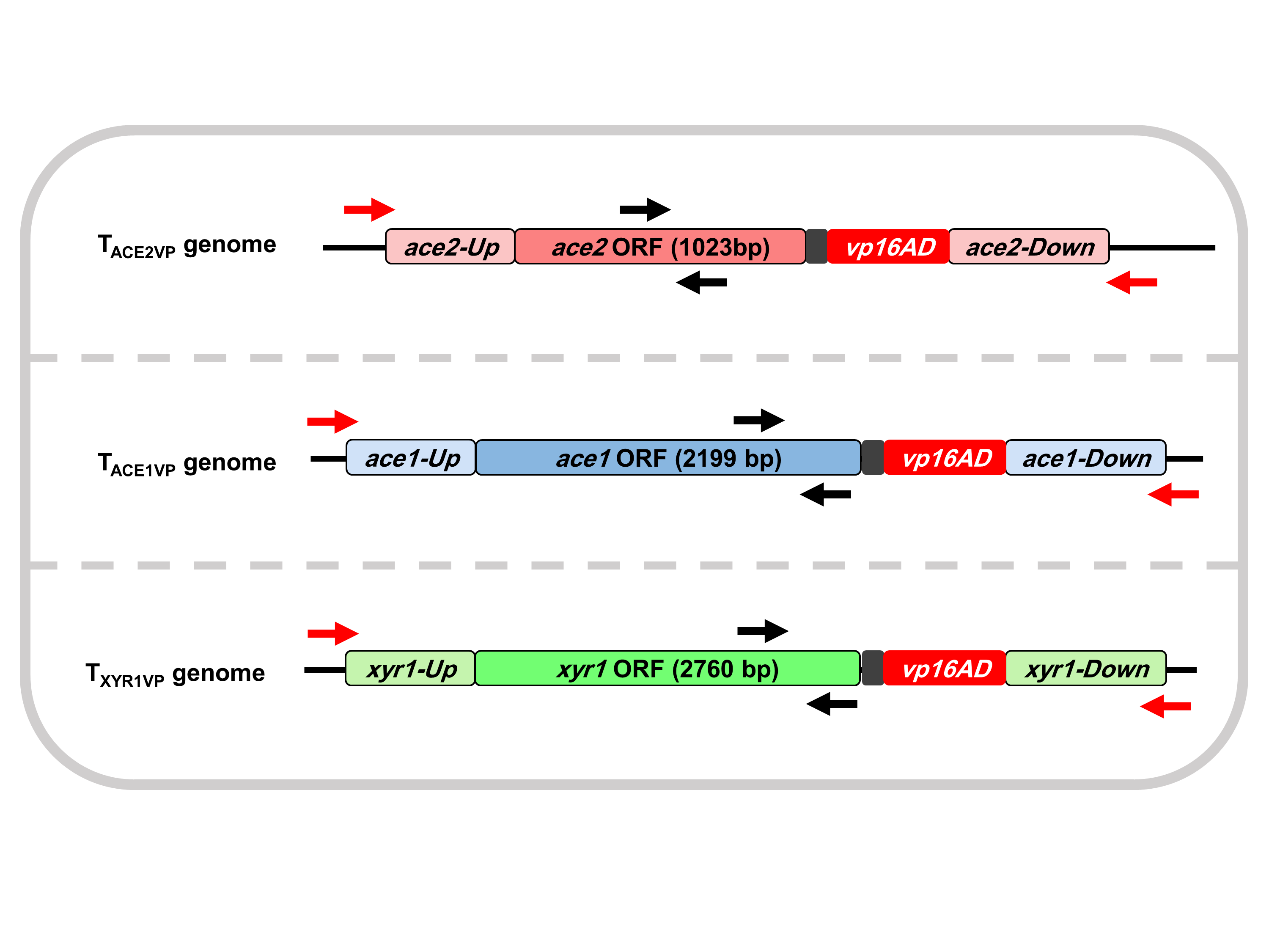
**

**Fig. S1**

**
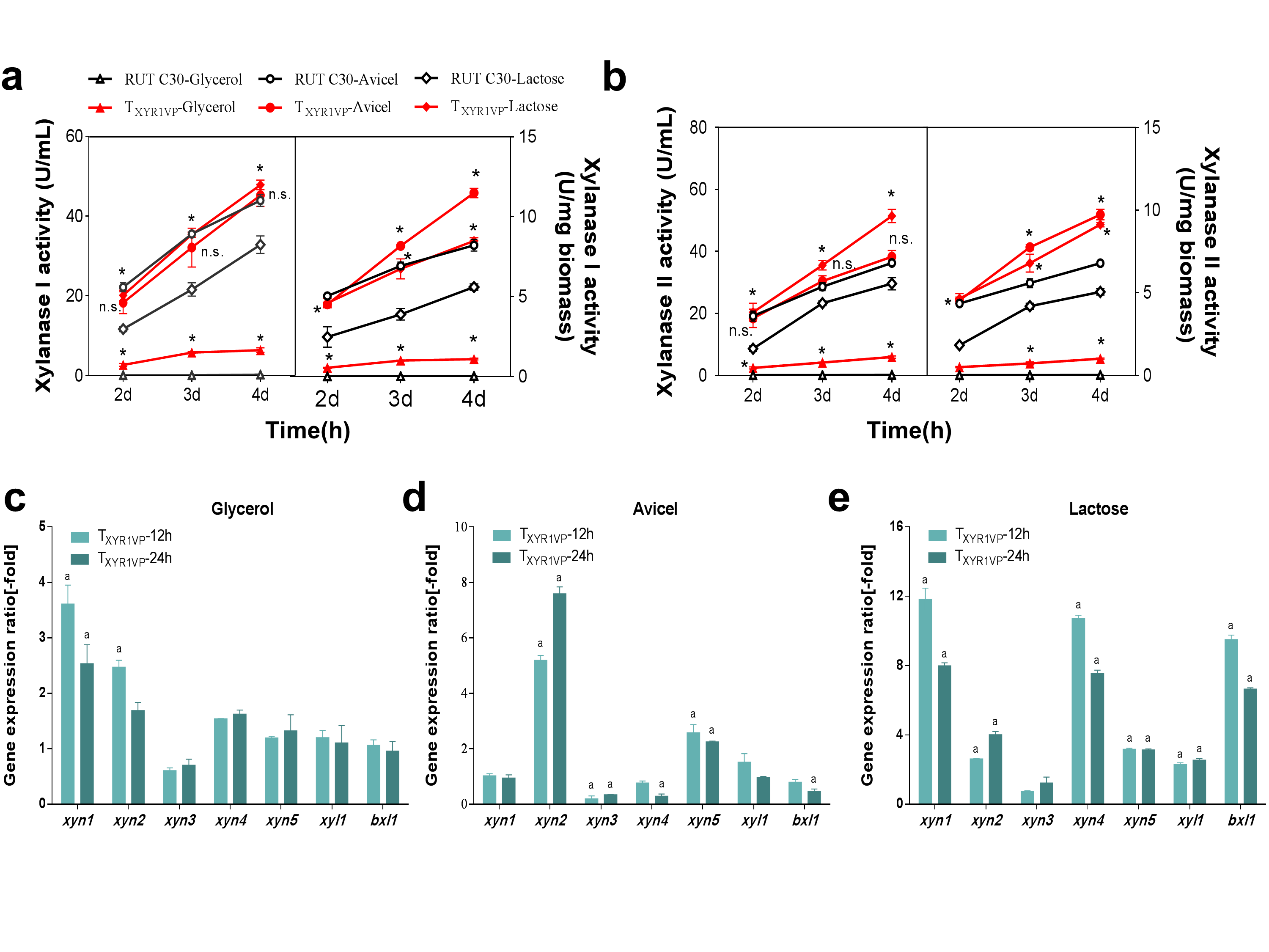
**

**Fig. S2**

**
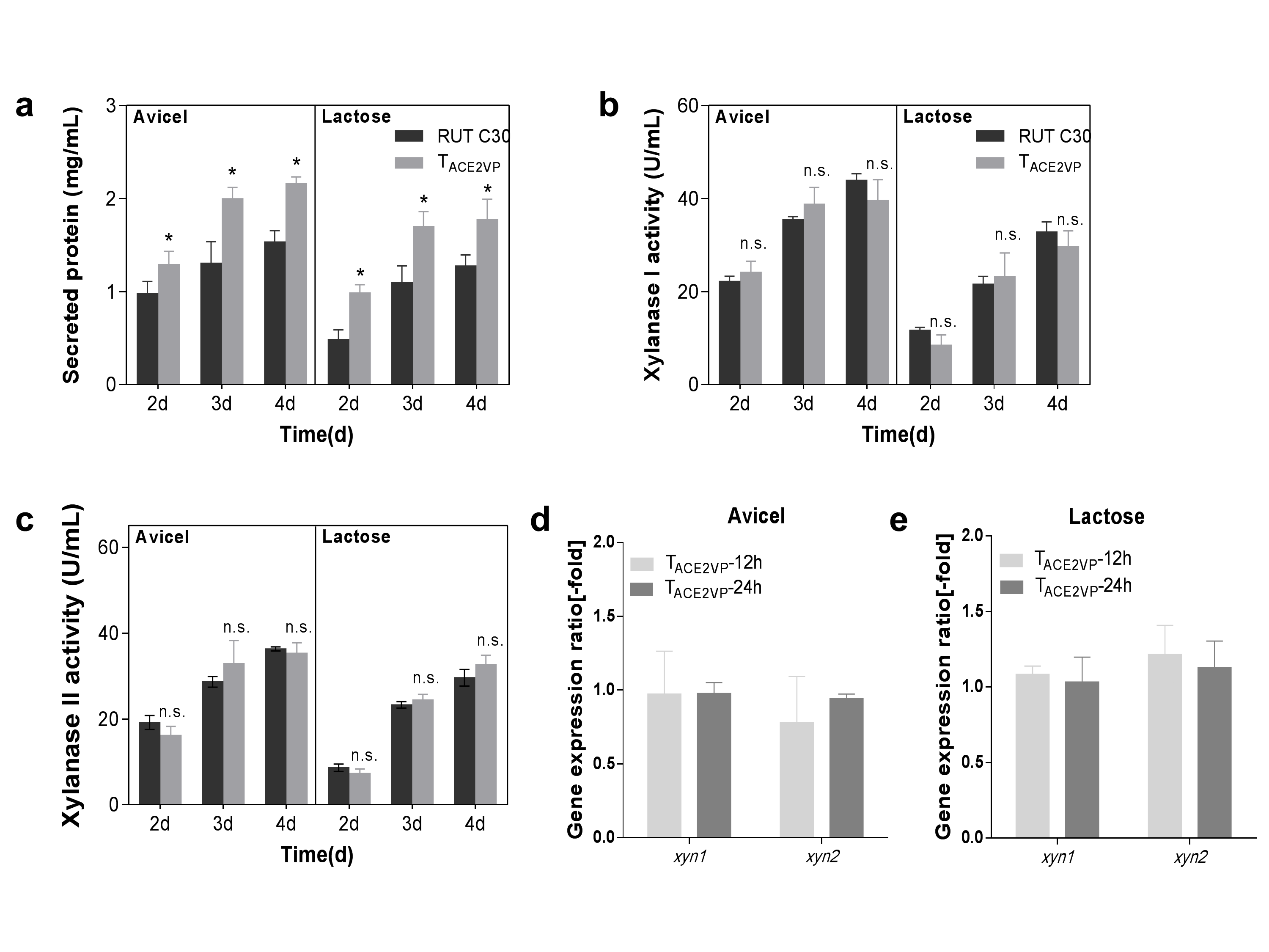
**

**Fig. S3**

**
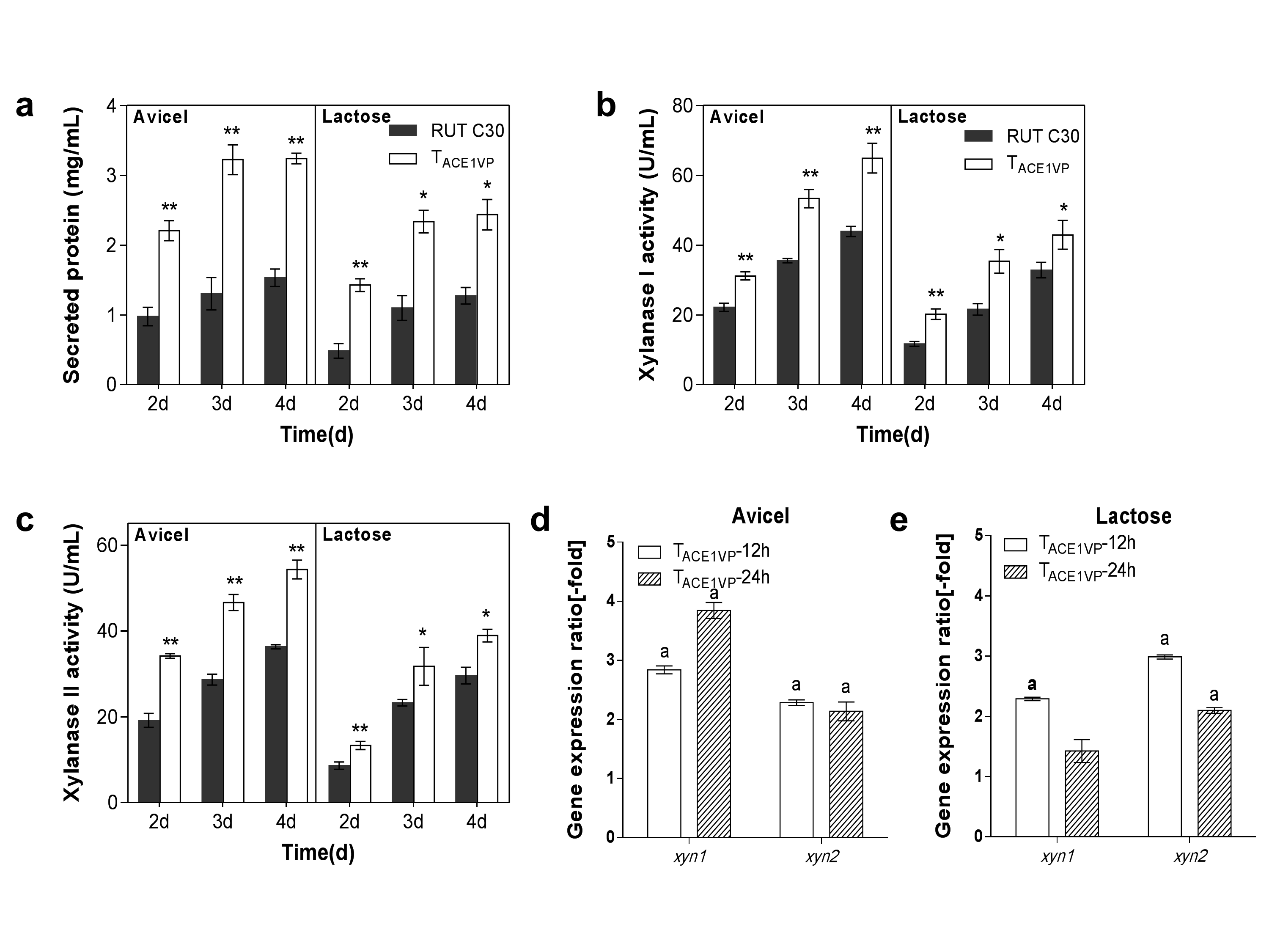
**

**Fig. S4**

**
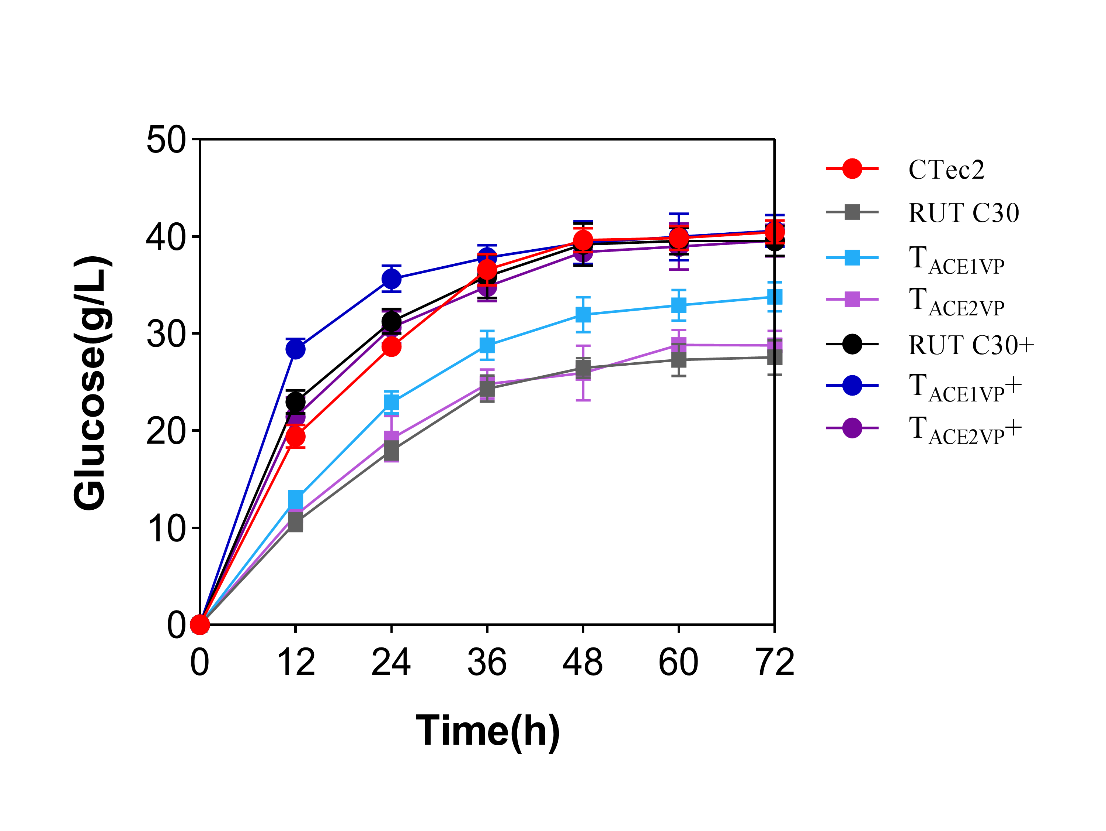
**

**Fig. S5**
